# 4-Eth­oxy-*N*′-propanoylpyridine-2-carbohydrazide

**DOI:** 10.1107/S1600536810010135

**Published:** 2010-03-27

**Authors:** Sheng-Jiao Tang, Hai-Ping Li, Xin-Yong Lin, Wen-Shi Wu

**Affiliations:** a College of Materials Science and Engineering, Huaqiao University, Xiamen, Fujian 361021, People’s Republic of China

## Abstract

In the crystal structure of the title compound, C_11_H_15_N_3_O_3_, mol­ecules are linked into a chain by inter­molecular N—H⋯O hydrogen bonds.

## Related literature

For the structure of *N*-propionylpicoloylhydrazide, see: Wu & Liu (2001 ▶). 
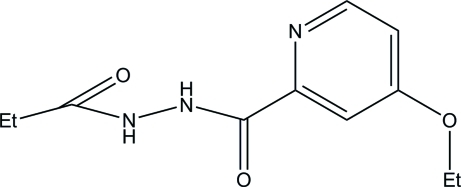


## Experimental

### 

#### Crystal data

C_11_H_15_N_3_O_3_
*M* *_r_* = 237.26
Monoclinic, 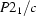

*a* = 11.377 (5) Å
*b* = 4.745 (2) Å
*c* = 23.244 (10) Å
β = 99.534 (5)°
*V* = 1237.3 (9) Å^3^
*Z* = 4
Mo *K*α radiation
μ = 0.09 mm^−1^
*T* = 293 K
1.00 × 0.45 × 0.10 mm

#### Data collection

Bruker P4 diffractometer
9032 measured reflections
2803 independent reflections
2297 reflections with *I* > 2σ(*I*)
*R*_int_ = 0.022

#### Refinement

*R*[*F* ^2^ > 2σ(*F* ^2^)] = 0.053
*wR*(*F* ^2^) = 0.150
*S* = 0.96
2803 reflections
154 parameters
H-atom parameters constrained
Δρ_max_ = 0.27 e Å^−3^
Δρ_min_ = −0.28 e Å^−3^

### 

Data collection: *XSCANS* (Bruker, 1999 ▶); cell refinement: *XSCANS*; data reduction: *SHELXTL* (Sheldrick, 2008 ▶); program(s) used to solve structure: *SHELXS97* (Sheldrick, 2008 ▶); program(s) used to refine structure: *SHELXL97* (Sheldrick, 2008 ▶); molecular graphics: *SHELXTL*; software used to prepare material for publication: *SHELXTL*.

## Supplementary Material

Crystal structure: contains datablocks I, global. DOI: 10.1107/S1600536810010135/jh2133sup1.cif

Structure factors: contains datablocks I. DOI: 10.1107/S1600536810010135/jh2133Isup2.hkl

Additional supplementary materials:  crystallographic information; 3D view; checkCIF report

## Figures and Tables

**Table 1:** Hydrogen-bond geometry (Å, °)

*D*—H⋯*A*	*D*—H	H⋯*A*	*D*⋯*A*	*D*—H⋯*A*
N2—H2*A*⋯O2^i^	0.86	2.43	3.067 (2)	132
N3—H3*A*⋯O2^ii^	0.86	2.06	2.831 (2)	150

Symmetry codes: (i) 
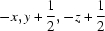
; (ii) 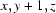.

**Fig. 1.**
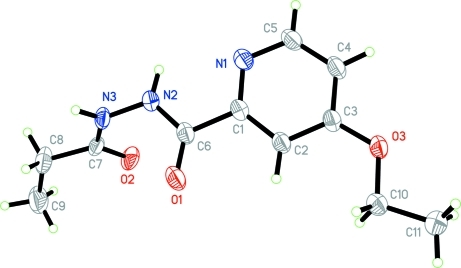
View of the title compound showing the atom-labelling scheme. Displacement ellipsoids are drawn at the 30% probability level. H atoms are represented by circles of arbitrary radius.

**Fig. 2.**
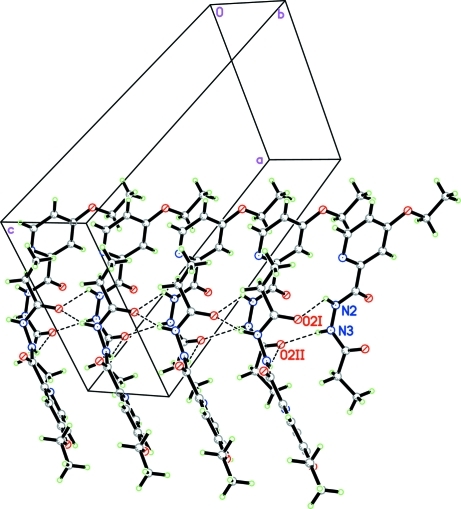
Hydrogen bonds diagram of the title compound, showing the H-bonded interactions (dashed lines). O2I, O2II represent O2i, O2ii, respectively.

## supplementary crystallographic information

### Comment

It has been reported that the structure of N-propionylpicoloylhydrazide (II, Wu *et al.*, 2001), based on which we reported the structure of 4-ethoxy-*N*-propionyl-2-pyridine formylhydrazine (I), C_11_H_15_N_3_O_3_. The structure of the title compound shown in Fig. 1 exhibits a stable one-dimension chain structure which is stabilized by inter-molecular hydrogen bonds of N2—H2A···O2^i^, N3—H3A···O2^ii^. All these bonds are detailed in Fig. 2 and Table 1.

In this title compound, the torsion angle of C6—N2—N3—C7 is -77.1 (2) ° which is slightly smaller than the torsion angle of the structure (II) (-73.5 (2) ° for C6—N2—N3—C7). The distances of C6—N2, N2—N3 and C7—N3 are 1.345 (2) Å, 1.335 (2) and 1.380 (2) Å respectively. They are similar to homologous bonds of structure (II) with distances 1.334 (2) Å for C6—N2, 1.383 (2) Å for N2—N3 and 1.337 (2) Å for C7—N3. And in structure (I), it's almost coplanar for C1, C2, C3, C4, C5, N1, C6, O1, N2, N3, O3, C10 and C11, and the maximum atomic deviation being 0.0920 Å. The dihedral angle between the mean planes of the C1, C2, C3, C4, C5, N1, C6, O1, N2, N3, O3, C10 and C11 and the mean planes of the N3, C7 and O2 is 72.44 (8) °.

### Experimental

4-ethoxyl-2-pyridine formylhydrazine (3.42 g, 14.41 mmol) was dissolved in a mixed solution of CHCl_3_ (30 ml) and EtOH (20 ml), then filtered. Propionic anhydride (3.64 ml) was added and refluxed 2hrs with whisked. Colorless needle crystals of the title compound were obtained by slow evaporation of solvent at room temperature. Melting point: 407 - 407.5 K.

### Refinement

The positions of the N2-, N3-bound H atoms were placed at fixed positions and refined accord to the riding model. The C-bound H atoms were included in the riding model approximation with C—H = 0.93 Å and U_iso_ of each H atom = 1.2U_eq_(C).

### Crystal data

**Table:**

C_11_H_15_N_3_O_3_	*F*(000) = 504
*M**_r_* = 237.26	*D*_x_ = 1.274 Mg m^−^^3^
Monoclinic, *P*2_1_/*c*	Melting point = 407–407.5 K
Hall symbol: -P 2ybc	Mo *K*α radiation, λ = 0.71073 Å
*a* = 11.377 (5) Å	Cell parameters from 2827 reflections
*b* = 4.745 (2) Å	θ = 2.7–27.5°
*c* = 23.244 (10) Å	µ = 0.09 mm^−^^1^
β = 99.534 (5)°	*T* = 293 K
*V* = 1237.3 (9) Å^3^	Prism, colourless
*Z* = 4	1.00 × 0.45 × 0.10 mm

### Data collection

**Table:**

Bruker P4 diffractometer	2297 reflections with *I* > 2σ(*I*)
Radiation source: fine-focus sealed tube	*R*_int_ = 0.022
graphite	θ_max_ = 27.5°, θ_min_ = 2.7°
Detector resolution: 0 pixels mm^-1^	*h* = −14→11
ω scans	*k* = −6→6
9032 measured reflections	*l* = −24→30
2803 independent reflections	

### Refinement

**Table:**

Refinement on *F*^2^	Primary atom site location: structure-invariant direct methods
Least-squares matrix: full	Secondary atom site location: difference Fourier map
*R*[*F*^2^ > 2σ(*F*^2^)] = 0.053	Hydrogen site location: inferred from neighbouring sites
*wR*(*F*^2^) = 0.150	H-atom parameters constrained
*S* = 0.96	*w* = 1/[σ^2^(*F*_o_^2^) + (0.080*P*)^2^ + 0.3962*P*] where *P* = (*F*_o_^2^ + 2*F*_c_^2^)/3
2803 reflections	(Δ/σ)_max_ < 0.001
154 parameters	Δρ_max_ = 0.27 e Å^−^^3^
0 restraints	Δρ_min_ = −0.28 e Å^−^^3^

### Special details

**Table:**

Geometry. All esds (except the esd in the dihedral angle between two l.s. planes) are estimated using the full covariance matrix. The cell esds are taken into account individually in the estimation of esds in distances, angles and torsion angles; correlations between esds in cell parameters are only used when they are defined by crystal symmetry. An approximate (isotropic) treatment of cell esds is used for estimating esds involving l.s. planes.
Refinement. Refinement of *F*^2^ against ALL reflections. The weighted *R*-factor wR and goodness of fit *S* are based on *F*^2^, conventional *R*-factors *R* are based on *F*, with *F* set to zero for negative *F*^2^. The threshold expression of *F*^2^ > σ(*F*^2^) is used only for calculating *R*-factors(gt) etc. and is not relevant to the choice of reflections for refinement. *R*-factors based on *F*^2^ are statistically about twice as large as those based on *F*, and *R*- factors based on ALL data will be even larger.

### Fractional atomic coordinates and isotropic or equivalent isotropic displacement parameters (Å^2^)

**Table:**

	*x*	*y*	*z*	*U*_iso_*/*U*_eq_	
C1	0.16780 (13)	0.4979 (3)	0.41169 (7)	0.0451 (4)	
C2	0.18448 (13)	0.3174 (3)	0.45907 (7)	0.0476 (4)	
H2	0.1211	0.2618	0.4771	0.057*	
C3	0.29993 (13)	0.2221 (4)	0.47874 (6)	0.0467 (4)	
C4	0.39038 (13)	0.3146 (4)	0.45039 (7)	0.0552 (4)	
H4	0.4682	0.2531	0.4622	0.066*	
C5	0.36375 (14)	0.4981 (4)	0.40462 (8)	0.0608 (5)	
H5	0.4260	0.5619	0.3867	0.073*	
C6	0.04284 (13)	0.5972 (4)	0.38881 (7)	0.0493 (4)	
C7	−0.15075 (13)	0.6370 (3)	0.27838 (7)	0.0446 (4)	
C8	−0.26718 (18)	0.7610 (4)	0.24930 (10)	0.0732 (6)	
H8A	−0.2938	0.8948	0.2760	0.088*	
H8B	−0.2530	0.8654	0.2152	0.088*	
C9	−0.36264 (19)	0.5625 (6)	0.23120 (15)	0.1004 (9)	
H9D	−0.4327	0.6624	0.2134	0.151*	
H9A	−0.3795	0.4607	0.2646	0.151*	
H9B	−0.3391	0.4325	0.2036	0.151*	
C10	0.24191 (15)	−0.0530 (4)	0.55627 (8)	0.0557 (4)	
H10A	0.1822	−0.1624	0.5311	0.067*	
H10B	0.2029	0.1047	0.5718	0.067*	
C11	0.30482 (18)	−0.2325 (5)	0.60472 (9)	0.0708 (6)	
H11A	0.2481	−0.3041	0.6274	0.106*	
H11B	0.3636	−0.1216	0.6292	0.106*	
H11C	0.3431	−0.3871	0.5886	0.106*	
N1	0.25346 (11)	0.5917 (3)	0.38402 (6)	0.0540 (4)	
N2	0.03317 (11)	0.7407 (3)	0.33833 (6)	0.0506 (3)	
H2A	0.0960	0.7822	0.3240	0.061*	
N3	−0.07761 (11)	0.8213 (3)	0.30948 (6)	0.0490 (3)	
H3A	−0.0999	0.9937	0.3116	0.059*	
O1	−0.04003 (11)	0.5488 (4)	0.41406 (6)	0.0787 (5)	
O2	−0.12542 (11)	0.3873 (2)	0.27472 (6)	0.0604 (4)	
O3	0.33191 (10)	0.0459 (3)	0.52425 (5)	0.0598 (4)	

### Atomic displacement parameters (Å^2^)

**Table:**

	*U*^11^	*U*^22^	*U*^33^	*U*^12^	*U*^13^	*U*^23^
C1	0.0370 (7)	0.0495 (8)	0.0482 (8)	0.0069 (6)	0.0051 (6)	−0.0025 (6)
C2	0.0360 (7)	0.0558 (9)	0.0511 (8)	0.0084 (6)	0.0075 (6)	0.0029 (7)
C3	0.0396 (7)	0.0533 (9)	0.0463 (8)	0.0093 (6)	0.0044 (6)	0.0002 (7)
C4	0.0325 (7)	0.0737 (11)	0.0576 (9)	0.0083 (7)	0.0025 (6)	0.0038 (8)
C5	0.0358 (8)	0.0857 (13)	0.0609 (10)	0.0011 (8)	0.0083 (7)	0.0104 (9)
C6	0.0391 (8)	0.0530 (9)	0.0554 (9)	0.0111 (7)	0.0062 (6)	0.0044 (7)
C7	0.0503 (8)	0.0319 (7)	0.0501 (8)	0.0043 (6)	0.0037 (6)	0.0105 (6)
C8	0.0658 (11)	0.0457 (9)	0.0953 (15)	0.0063 (8)	−0.0247 (10)	0.0110 (9)
C9	0.0567 (12)	0.0702 (14)	0.162 (3)	0.0015 (10)	−0.0182 (14)	0.0214 (15)
C10	0.0455 (8)	0.0638 (10)	0.0591 (10)	0.0094 (7)	0.0124 (7)	0.0056 (8)
C11	0.0607 (11)	0.0857 (14)	0.0659 (11)	0.0081 (10)	0.0104 (9)	0.0210 (10)
N1	0.0393 (7)	0.0669 (9)	0.0549 (8)	0.0039 (6)	0.0054 (6)	0.0076 (7)
N2	0.0378 (6)	0.0494 (7)	0.0627 (8)	0.0066 (5)	0.0027 (6)	0.0113 (6)
N3	0.0445 (7)	0.0314 (6)	0.0666 (8)	0.0084 (5)	−0.0034 (6)	0.0054 (6)
O1	0.0440 (7)	0.1159 (12)	0.0797 (9)	0.0281 (7)	0.0204 (6)	0.0354 (8)
O2	0.0739 (8)	0.0305 (6)	0.0743 (8)	0.0092 (5)	0.0053 (6)	0.0045 (5)
O3	0.0407 (6)	0.0781 (8)	0.0612 (7)	0.0186 (5)	0.0099 (5)	0.0205 (6)

### Geometric parameters (Å, °)

**Table:**

C1—N1	1.330 (2)	C8—C9	1.446 (3)
C1—C2	1.383 (2)	C8—H8A	0.9700
C1—C6	1.508 (2)	C8—H8B	0.9700
C2—C3	1.393 (2)	C9—H9D	0.9600
C2—H2	0.9300	C9—H9A	0.9600
C3—O3	1.3502 (19)	C9—H9B	0.9600
C3—C4	1.382 (2)	C10—O3	1.440 (2)
C4—C5	1.369 (3)	C10—C11	1.496 (3)
C4—H4	0.9300	C10—H10A	0.9700
C5—N1	1.342 (2)	C10—H10B	0.9700
C5—H5	0.9300	C11—H11A	0.9600
C6—O1	1.212 (2)	C11—H11B	0.9600
C6—N2	1.345 (2)	C11—H11C	0.9600
C7—O2	1.2256 (19)	N2—N3	1.3800 (17)
C7—N3	1.335 (2)	N2—H2A	0.8600
C7—C8	1.504 (2)	N3—H3A	0.8600
			
N1—C1—C2	125.26 (14)	C8—C9—H9D	109.5
N1—C1—C6	116.63 (14)	C8—C9—H9A	109.5
C2—C1—C6	118.10 (14)	H9D—C9—H9A	109.5
C1—C2—C3	117.38 (14)	C8—C9—H9B	109.5
C1—C2—H2	121.3	H9D—C9—H9B	109.5
C3—C2—H2	121.3	H9A—C9—H9B	109.5
O3—C3—C4	116.42 (13)	O3—C10—C11	106.39 (14)
O3—C3—C2	125.11 (14)	O3—C10—H10A	110.5
C4—C3—C2	118.46 (15)	C11—C10—H10A	110.5
C5—C4—C3	119.08 (14)	O3—C10—H10B	110.5
C5—C4—H4	120.5	C11—C10—H10B	110.5
C3—C4—H4	120.5	H10A—C10—H10B	108.6
N1—C5—C4	124.12 (16)	C10—C11—H11A	109.5
N1—C5—H5	117.9	C10—C11—H11B	109.5
C4—C5—H5	117.9	H11A—C11—H11B	109.5
O1—C6—N2	124.08 (14)	C10—C11—H11C	109.5
O1—C6—C1	122.24 (15)	H11A—C11—H11C	109.5
N2—C6—C1	113.69 (14)	H11B—C11—H11C	109.5
O2—C7—N3	122.59 (14)	C1—N1—C5	115.67 (15)
O2—C7—C8	123.14 (15)	C6—N2—N3	120.02 (13)
N3—C7—C8	114.25 (13)	C6—N2—H2A	120.0
C9—C8—C7	116.04 (16)	N3—N2—H2A	120.0
C9—C8—H8A	108.3	C7—N3—N2	121.25 (12)
C7—C8—H8A	108.3	C7—N3—H3A	119.4
C9—C8—H8B	108.3	N2—N3—H3A	119.4
C7—C8—H8B	108.3	C3—O3—C10	118.97 (12)
H8A—C8—H8B	107.4		
			
N1—C1—C2—C3	−1.2 (3)	N3—C7—C8—C9	−161.1 (2)
C6—C1—C2—C3	178.43 (14)	C2—C1—N1—C5	0.4 (3)
C1—C2—C3—O3	179.96 (15)	C6—C1—N1—C5	−179.20 (15)
C1—C2—C3—C4	0.6 (2)	C4—C5—N1—C1	1.0 (3)
O3—C3—C4—C5	−178.73 (17)	O1—C6—N2—N3	−5.8 (3)
C2—C3—C4—C5	0.7 (3)	C1—C6—N2—N3	173.92 (13)
C3—C4—C5—N1	−1.6 (3)	O2—C7—N3—N2	1.7 (2)
N1—C1—C6—O1	−172.29 (17)	C8—C7—N3—N2	−179.35 (16)
C2—C1—C6—O1	8.0 (3)	C6—N2—N3—C7	−77.1 (2)
N1—C1—C6—N2	8.0 (2)	C4—C3—O3—C10	178.74 (16)
C2—C1—C6—N2	−171.66 (14)	C2—C3—O3—C10	−0.7 (3)
O2—C7—C8—C9	17.9 (3)	C11—C10—O3—C3	−177.84 (16)

### Hydrogen-bond geometry (Å, °)

**Table:**

*D*—H···*A*	*D*—H	H···*A*	*D*···*A*	*D*—H···*A*
N2—H2A···O2^i^	0.86	2.43	3.067 (2)	132
N3—H3A···O2^ii^	0.86	2.06	2.831 (2)	150

Symmetry codes: (i) −*x*, *y*+1/2, −*z*+1/2; (ii) *x*, *y*+1, *z*.
